# The Use of Traditional Korean Medicine (TKM) by Children: A Correlational Study between Parent’s Perception and Their Children’s Use Reported by Parents

**DOI:** 10.3390/healthcare9040385

**Published:** 2021-04-01

**Authors:** Jihye Kim, Jang-Kyung Park, Jung-Youn Park, Eun-Jin Lee, Soo-Hyun Sung

**Affiliations:** 1 Research Institute of Korean Medicine Policy, The Association of Korean Medicine, Seoul 07525, Korea; jihyekim1217@gmail.com; 2 Department of Obstetrics and Gynecology, College of Korean Medicine, Pusan National University, Yangsan 50612, Korea; vivat314@pusan.ac.kr; 3 Department of Health and Welfare, Yuhan University, Bucheon 14780, Korea; park0625@yuhan.ac.kr; 4 Department of Policy Development, National Development Institute of Korean Medicine, Seoul 04554, Korea; eunjin6434@nikom.or.kr

**Keywords:** traditional Korean medicine (TKM), complementary and alternative medicine (CAM), national survey TKM usage, parents’ perception of TKM, children’s TKM use

## Abstract

This cross-sectional study investigated the correlation between parents’ perception and their children’s traditional Korean medicine (TKM) use reported by parents in order to discover policy intervention points and provide a reference for establishing generalized TKM policies. Participant data from a 2017 national survey on TKM usage was divided into two groups based on the children’s TKM use reported by parents. The female participants’ children had a higher rate of experience in using TKM (8.1%; *p* = 0.029). Additionally, 91.4% of the parent group with a child who used TKM turned out to have used TKM, which was higher than 71.9% of the parents whose children never used TKM (*p* < 0.001). As for the awareness on the use of TKM, 44.0% of the parents with a child who experienced TKM answered they were aware of it, while only 35.3% of the parent group whose child never experienced TKM did so (*p* = 0.033). The present study suggests that parental experience in using TKM could have an impact on the children’s TKM use reported by parents. Further study is necessary to assess which parental factor (awareness level, medical disorder to be treated, therapy, therapeutic efficacy, the purpose of visit, sex, age, etc.) has a close relationship with TKM usage experience of their children.

## 1. Introduction

Complementary and Alternative Medicine (CAM), which is not considered to be part of conventional medicine, is a compilation of knowledge, skills, and practices which are based on the theories, beliefs, and experiences indigenous to different cultures and used for health maintenance and in the prevention, diagnosis, improvement, or treatment of physical and mental illness [1,2,3]. CAM approaches include natural products (e.g., herbs, vitamins and minerals and probiotics), and mind body practice such as yoga, meditation, chiropractic, acupuncture, relaxation techniques, tai chi, qigong, and hypnotherapy [4]. The use of CAM is increasing in many countries of the world [5]. Approximately 88% of the member states of the World Health Organization (WHO) are using CAM through the development of national policies, laws, regulations, and applied programs [6]. In East-Asian countries such as South Korea and China, traditional medicine has been the form of medical care treating the diseases of the people. Currently, it is still taking a crucial part in health care along with conventional medicine (CM) [7,8]. In East-Asian countries such as China, Korea, and Taiwan, traditional medicine practitioners are considered as doctors, as is the case with the doctors who provide CM [9,10]. Traditional Korean medicine (TKM) doctors use acupuncture, electro-acupuncture, pharmacopuncture, herbal medicine, chuna, cupping, moxibustion, and other forms of intervention to treat their patients [11]. In Korea, 14% of the total population and 7.6% of those in their 20 s or younger, are using TKM, and 10% of the total male population and 18.1% of the total female population visited TKM clinics [12]. The purpose of using TKM was treating a disease (94.1%), improving health (18.4%), and cosmetic purposes (4.0%) [13].

Health in the pediatric or juvenile period has an impact on adulthood health, education, achievement, and economic performance. Therefore, the health of children and teenagers is of paramount importance [14,15]. Additionally, parents’ experience with using medical services can have varying effects on their children [16]. Some well-known factors impacting the health and the usage of medical services during pediatric and juvenile periods include respective family structure, parental education level, and their social-economic status [17,18,19,20,21,22].

In the field of traditional Chinese medicine (TCM), Loh [23] surveyed 300 parents who visited TCM clinics, and 84.3% of their children used TCM clinics, while 80.3% of them reported that they used both TCM clinics and CM clinics. Yeh [24] used the National Health Insurance data of Taiwan to analyze the overall usage of TCM and reported that about 20% of the children under 20 used TCM clinics. In the field of TKM, Choi [25] conducted a survey of 300 parents who used the TKM clinic and had a child under 19. As a result of the said survey, it was resulted that 81% of the children experienced and visited TKM clinics for the purpose of treating respiratory disease (21.6%) gastrointestinal disease (10.6%,), and skin disease (9.2%). Park [26] surveyed 702 parents who used daycare centres and reported that 55.3% of the children’s age from 1 to 13 used TKM clinics, mainly for the purpose of treating respiratory disease (34.5%), gastrointestinal disease (17.2%), and skin disease (13.8%). As such, the previous studies on the usage of traditional medicine among children or adolescents under 20, were mainly conducted as cross-sectional studies. In three studies [23,25,26], the study samples were not representative of the general population of the country. Yeh [24] used the representative data of the general population of Taiwan. However, the study was intended to investigate the usage of TCM in the entire population, including children.

An assessment of TKM usage has been undertaken by Statistics Korea, as a certified national statistic every 3 years since 2008. This assessment covers all household members of the sample families who are at least 19 years old, providing a representative sample of the country [13]. In order to effectively integrate TKM into the healthcare system, it is necessary that policies are developed and implemented based on accurate statistics of TKM. To fulfil this requirement, the national survey on TKM use data from 5000 Korean participants was used in order to examine the correlation between parental awareness of TKM use and the use of TKM by their children.

## 2. Materials and Methods

### 2.1. Data Sources

The source data used in this study is from the TKM usage survey of 2017, conducted by the Ministry of Health and Welfare and National Institute of Korean Medicine Development. The survey was reviewed and approved by Korea Statistics (National Certified Statistics Approval Number: 117087). As for the survey data, the researchers made a request for micro-data through the surveys on the traditional herbal medicines consumption and the TKM usage database (https://www.koms.or.kr/main.do, accessed on 18 November 2020) run by the National Institute of Korean Medicine Development (NIKOM). Approval was obtained to use and analyze the data from the NIKOM, which was commissioned by the government to conduct the above-mentioned survey and analyzed the micro-data which was provided by the said organization to conduct this study.

As for the surveys on the traditional herbal medicine consumption and the TKM usage, the survey on the usage of TKM started in 2008 (1st survey), which was followed by the survey on the consumption of traditional herbal medicine (1st survey) in 2009. In 2011, the two separate surveys were integrated into one survey on TKM usage. Since then, the survey has been conducted every 3 years. Among all the data collected over the years, we used the data on the usage of TKM obtained during the 4th survey that took place in 2017.

### 2.2. Sample Selection

**Figure 1:**
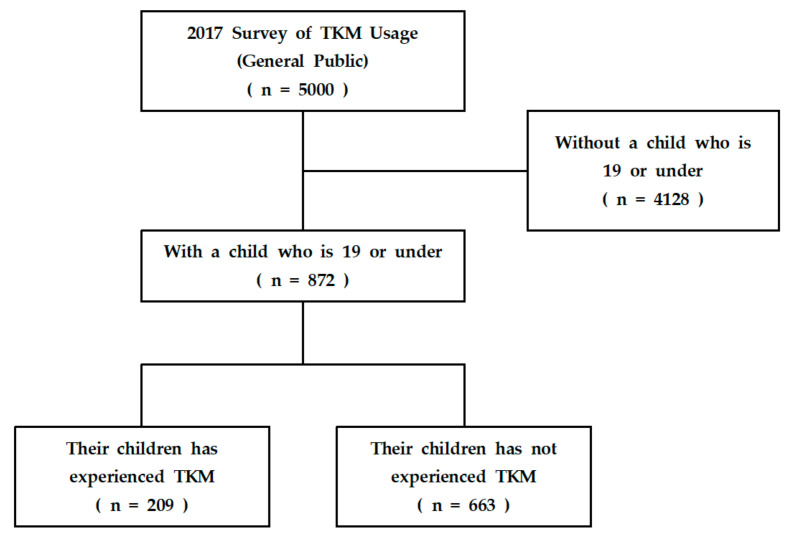
Flow chart of inclusion and exclusion of participants from the 2017 National Survey of Traditional Korean Medicine Usage. TKM: Traditional Korean Medicine.

The data used in this study was obtained from 5000 participants selected among the general population during the TKM usage survey in 2017. When the 5000 participants, who were the national survey samples, were selected, they did not enroll both parents from a family. In particular the following selecting questions were used to assess eligibility: Do you have a child who is under 19 (born after September 1998)? (1) Yes [the number of children under 19 is (…)] (2) No. The participants in the survey item or were the parent who was the caregiver of the children. Also, we instructed the participants to tell us the number of children in their family if they answered they had a child under 19. This resulted in the inclusion of 872 participants who chose ‘Yes’. Their answers were included for analysis in order to obtain the results on the satisfaction and awareness data, as well as the information regarding their children. The selection process of the participants is shown in Figure 1.

### 2.3. Analysis Items

The analysis items included in this study were the participants who answered ‘Yes’ to the question asking whether they had a child under the age of 19, during the TKM usage survey in 2017, as well as the questions regarding the satisfaction and awareness of their children. Additionally, based on the children’s TKM use reported by parents, the relationships between the demographics, the experience of using TKM, their opinion on the use of TKM, and the perception of treatment effects on sixteen diseases, were analyzed. The demographics of the participants (mother or father) were used to examine the relationship between the answers about the children and the characteristics of participants. The answers about the children included the children’s experience with TKM, the reason why their children used TKM, the kind of TKM treatment the children experienced, the degree of satisfaction with their children’s TKM experience, and cause of dissatisfaction with using TKM. The basic demographic information of the participants included their sex, age group, area of residence, education, employment status, household income, type of healthcare coverage, and subscription status of private medical insurance. The experience of using TKM variables included TKM participation, the reason for choosing to use TKM, participants’ experience with TKM, participants’ satisfaction with TKM, participants’ awareness of TKM, money invested to use TKM, their willingness to use TKM again in the future, and their willingness to recommend TKM to others. Regarding the perception of treatment effects on diseases, variables were used following sixteen diseases: (1) disc related disease (herniation of intervertebral disc, spinal stenosis); (2) osteoarthritis; (3) frozen shoulder shoulder pain; (4) back pain; (5) sprain; (6) facial nerve paralysis; (7) stroke; (8) hypertension; (9) diabetes mellitus; (10) digestive disease; (11) common cold rhinitis; (12) dementia (13) cancer related pain; (14) infertility; (15) skin disease (atopic dermatitis); (16) genitourinary disease. Also, whether the characteristics of the participants (demographics, their opinion or ideas on TKM, etc.) had an impact on the children’s use of TKM and the possible strategies to promote use of TKM in different target groups, were explored.

### 2.4. Statistical Analysis

In order to better understand the general characteristics of the participants included for analysis, the frequencies and ratios were calculated. Based on the children’s TKM use reported by parents, the correlations between variables were analyzed using the chi-square test (χ^2^-test), which is a cross-analysis method of the ratios between different groups and is commonly used when analyzing categorized data. IBM SPSS Statistics for Windows, version 25 (IBM Corp., Armonk, NY, USA) was used for all statistical analyses and the significance level was set at 5% (*p* < 0.05).

## 3. Results

### 3.1. The Information on and Characteristics of the Children of the Participants

The basic statistics of the 5000 participants (fathers or mothers) from the general population who participated in the survey were obtained based on their answers to the question regarding their children (Table 1). Regarding the question about their children, 872 participants answered that they had a child (or children) under the age of 19, accounting for 17.4% of all participants (Table 1). The majority of these contributors answered that they only had one child that fell into that particular age group (*n* = 425, 48.7%), which was followed by those with two children in the corresponding age group (*n* = 393, 45.1%). When asked whether their children had any experience of using TKM over the past 12-month period, 209 answered they had, while 663 answered none (24.0% and 76.0%, respectively).

### 3.2. The Demographics of the Parents and Their Children’s TKM Use Reported by Parents

Differences in the children’s demographics were assessed based on their (or their parents) experience with TKM (Table 2). Interestingly, female participants were more likely to have children with experience of using TKM (*p* = 0.029). Furthermore, the chance of having a child with experience of using TKM was higher in Chungcheong (*n* = 116, 13.3%) and Gyeongsang (*n* = 225, 25.8%) areas compared to the capital area (*n* = 371, 42.5%). Participants in their 50 s (50 to 60 years or older) showed a higher value compared to participants that were 40 years old or younger (30 to 40 years or younger) (*p* = 0.042). There was also no difference in employment status in terms of the children’s TKM usage reported by parents. No tendency of significance was observed with the household income as well. Participants with higher education levels (university or higher education) were more likely to answer that their children had experienced using TKM. However, the difference was not statistically significant. As for the types of government health care coverage, the groups without a child who experienced using TKM had a higher rate of having workplace health insurance coverage. Similarly, the group with children with experience of using TKM had a higher rate of having private medical insurance. However, these differences were non-statistically significant. Additionally, no significant differences were observed based on household income or area of residence.

### 3.3. The Opinion of the Parents on Using TKM and Their Children’s TKM Use Reported by Parents

Table 3 shows whether the children’s TKM use reported by parents changed depending on the parents’ ideas or experience in using TKM. This analysis revealed that the group with a child having experienced using TKM had a higher rate of having experienced TKM themselves, compared with the group without a child who experienced TKM (*p* < 0.001). Overall, the participants’ satisfaction with the TKM did not show a significant difference. Also, parents of the children with experience of using TKM showed a higher level of awareness of TKM (*p* = 0.033). The parents pre-conceived ideas regarding TKM did not result in a statistically significant result. The group with a child who experienced using TKM (*n* = 198, 94.7%) had a higher number of participants who answered that they were willing to use TKM in the future, compared to children who did not experience using TKM (*n* = 565, 85.2%) (*p* = 0.003). The group with children with experience of using TKM was more willing to recommend TKM to others (*p* < 0.001).

The analysis based on the education levels of the participants showed that, of the 191 participants where all the children and the parents experienced TKM, the proportion of those with higher education was higher than the proportion of the same among the participants where either of or all of the parents of the children had not experienced TKM. A similar trend could be observed in the answers to the question whether they are willing to revisit a TKM clinic or recommend it to others.

### 3.4. Perception of the TKM Effectiveness for Diseases and Their Children’s TKM Use Reported by Parents

Table 4 shows the analysis result on the perception of the treatment effectiveness for 16 diseases by the parents based on the children’s TKM use reported by parents. The group in which the children experienced TKM in 14 out of the 16 diseases showed a higher level of perception on the treatment effect for each of the diseases. Of these, the group where the children experienced TKM for two diseases showed a perception level on the treatment effect that was higher by 10% compared to the group in which the children never experienced TKM. The said difference was statistically significant (Common cold rhinitis: *p* = 0.011, skin disease: *p* = 0.002).

## 4. Discussion

In this study, which was based on the assumption that the experience, opinion, or ideas of parents may influence the use of TKM by their children, the participants were divided into groups based on whether their children had experienced using TKM or not; comparisons were made to evaluate whether these groups showed any differences in terms of the characteristics, awareness, or satisfaction of the parents. This analysis was done for the purpose of developing policies and identifying the ideal time for intervention by understanding the differences in satisfaction and awareness between the two groups.

The initial analysis regarding the participant’s children showed that, out of the 5000 participants, 17.4% (*n* = 872) had a child under the age of 19. Of these, 24% (*n* = 209) answered that their children experienced the use of TKM. In previous studies, it was reported that 55.3% of the target population had a child with TKM use experience [26], and in another, 81% [25]. However, caution should be practiced when interpreting these results as the target populations were from a daycare center within a self-governing district or the outpatients of a TKM clinic who were familiar with TKM pediatric practices. Especially, the value of 81% provided in Choi [25] was in contrast with the result of this study, where the proportion of the children who experienced TKM was 17.4%. It is believed to be because of the bias in the selection of the participants, who were selected from the patients who used the TKM clinic of the researcher. In this study, certified national statistics were used and thus based on a representative, standard sample of Korea’s general public; therefore, it is believed that the result can be generalized. Also, this study supports the findings in some preceding studies [25,26] that TKM clinics are used to treat the respiratory disease and skin disease in children. However, the said study could not cover the analysis on the purpose for the parents to use TKM clinics due to the limitations of the questions in the questionnaire, making it necessary to use caution in interpreting these findings, as it was assumed using the perception of treatment effect by parents based on the children’s TKM use reported by parents.

The reason why the children in Chungcheong and Gyeongsang regions are more likely to use TKM compared to those in the Capital area is attributed to the shortage of mainstream medical institutions (e.g., CM institutions and TKM institutions) and the demographics of these areas. These two regions are some of the most representative examples of a combination of urban and rural areas in the same region [27]. Also, the demographics of these areas are heavily leaning toward the older population compared to children [27].

The results of the analysis were used to observe whether there was a difference in the characteristics of the parents depending on the experience of their children with TKM showed that 8.1% more females (mothers) answered that their children had experienced using TKM. Also, as the participants grew older, being in their 50 s or older, the rate of answering that their children had experienced using TKM, tended to be higher. This supports the existing study result, where females were more likely to use TKM, and 61.8% of the users of TKM were at least 45 or older [28]. However, this study does not clarify the factors and correlations. Therefore, care is needed as one attempts to interpret it. Future studies are needed to investigate what is the true tendency of female and people in their 50 s or older in their use of TKM and what are the factors that contribute to such a result. Based on such findings, it would be possible to employ a more detailed approach to the use of TKM by the children.

On the other hand, the fact that those in their 30 s or 40 s were less likely to use TKM, compared to those in their 50 s or older, can be attributed to the fact that the younger group corresponds with the prime age of workers, namely those aged 25 to 49 [29], a time when individuals are the most active in terms of economic activities. In addition, if both parents of the children are working, it would be more likely that they would experience time or money obstacles, when trying to seek out using TKM. The findings of this study suggest that if the accessibility to TKM is improved for individuals in their 50 s, the younger parents in their 30 s to 40 s, and females, it would be possible to improve accessibility for their children. Also, in the group where the children had experienced TKM, the proportion of those with a higher level of education was higher among those who gave positive answers concerning the experience of the parents, intent to use in the future, and willingness to recommend. This can be interpreted to be because of the financial resources, desire to be healthy, and interest among these higher-education groups. However, further study is needed in order to clarify this correlation.

About 60% of the participants answered that TKM treatments were more expensive, which is related to the reimbursement ratio of the health care insurance of Korea. As of 2019, the reimbursement coverage rate by the health care insurance over the entirety of medical institutions in Korea was 64.2%, while the rate for TKM clinics was 54% and TKM hospitals was 28.7% [30]. This is based on the national policy that the health care insurance coverage is to be provided for the treatments with a clear scientific basis to treat severe diseases. While CM treatments have accumulated scientific evidence all over the world, CAM treatments and traditional treatments differ between countries, making it difficult to build up scientific evidence. Therefore, it is necessary to accumulate scientific evidence for the treatment effectiveness and safety by means of exchange and harmonization of traditional medicine and CAM with international organizations such as the World Health Organization taking the leading role. If such a worldwide basis of evidence is built, it could be possible to integrate traditional medicine and CAM treatments into the healthcare coverage, eventually contributing to the improvement of the health of the public.

Children of parents with an experience of using TKM were more likely to use TKM, compared with children whose parents had no experience with using TKM. Of the children whose parents experienced using TKM, 91.4% used TKM (*n* = 191) themselves, while 71.9% of the children whose parents never used TKM used it (*n* = 477) (*p* < 0.001). This indicates that the parents’ experience with using TKM had a significant impact (20%) on the use of TKM by their children. The Ministry of Health and Welfare developed the TKM health promotion program for toddlers and infants in 2016 [31], which has gradually been implemented through community health centers [32]. As such, in order for the State to maximize the effectiveness of childhood health management, mainly via TKM, a policy-based approach is needed such that the parents of these children may be provided with health management programs through TKM, as well.

In the group of parents whose children experienced using TKM, the rate of answering that they were willing to revisit a TKM clinic was higher by 9.5%, while the rate of answering that they were willing to recommend to others was higher by 20.8% (*p* = 0.003). This indicates that the perception of the parents impacted the use of TKM by their children, and the improvement of the perception of these parents may have an impact on the use of TKM by their children. It is necessary to conduct further studies in order to clarify these correlations.

The limitations of this study were as follows: first, due to the limitations in the data gathered for this study, it was not possible to clarify the correlations between the parents’ experience, awareness, and satisfaction with the use of TKM or the use of TKM by their children. Additional studies are needed in order to clarify which variables among the parents’ awareness, the purpose of visit, sex, or age, etc., had an impact on the use of TKM by their children. Second, the data obtained through the survey was based on the memories of parents, who were the participants of the survey. Therefore, it is still possible that they answered incorrectly when asked about their children’s experience with TKM. Third, the age group of the children of the participants could have had an impact on the experience of using a TKM clinic. However, it was not possible to obtain information on the age of the children. That is, younger children were more likely to have not experienced TKM, which could have contributed to the outcome. Lastly, due to the limitations in the questionnaires, it was not possible to identify the type of treatment the participating patients received. Among the CAM treatments [5,33], vitamins and minerals, probiotics, yoga, qigong, meditation, tai chi, relation techniques, and hypnotherapy are rarely used in TKM clinics, and health insurance coverage of TKM treatment [11] includes acupuncture, electro-acupuncture, pharmacopuncture, herbal medicine, chuna, cupping, and moxibustion. Therefore, it is difficult to compare the usage status and perception of TKM and CAM at the same level and generalize the results of this study.

The strength of this study is that it was conducted using data that is representative of the general Korean population, making the study more generalizable so that the study results can be used as a resource for the government to develop relevant policies. Also, it would be necessary to conduct an in-depth analysis on the decision-makers who decide which medical services to be used, as it is likely that the selection of the medical services used by a child is influenced by parental decisions.

In the future, the following strategies will be needed for the popularization of TKM. First, it is necessary to obtain precise statistical data on the factors and usage of TKM by improving the questionnaire items for the national survey in the future. Based on this study, it was possible to understand that the awareness of the family members on TKM could have an impact on the use of TKM by other members. However, due to the limitations in the question items in the questionnaire, we had difficulties in the analysis of correlations and factors. During the subsequent round of the national survey, the following supplementations are believed to be necessary: (1) Add questions regarding the experience of talking with a family physician or the experience of actually using the TKM for a disease or symptoms; (2) add more question items to ask whether the parents’ jobs were related to health care or they were actually healthcare professional, and if so, what type of healthcare professionals; (3) add more question items regarding awareness, the intent of use in the future, intent of the recommendation, and an item regarding the preference toward TKM; (4) more survey items for the children, regarding their age, education, and treated interventions and diseases; and (5) if questions are asked about the use of TKM by the children, conduct a face-to-face interview of the parents and the children at the same time. If the above-mentioned items are supplemented, it would be possible to clarify the point of intervention through policies by means of statistical analyses. Second, government-level standardization is required for the Clinical Practice Guideline (CPG) and the Clinical Pathway (CP) centered around the diseases for which TKM has an advantage over CM. Choose the diseases for which TKM has an advantage in different stages of the life cycle and develop corresponding CPGs and CPs (e.g., children; atopic dermatitis, females; dysmenorrhea, adults; back pain, seniors; osteoarthritis). With the policies to include these into the coverage of health care, it would have an impact on the family members of TKM users, contributing to popularization. In particular, the use and awareness of TKM by a female parent is likely to have an impact on her child. Therefore, it is necessary to survey the diseases in more details when it comes to female participants (e.g., menstruation, sub-fertility, post-natal management, climacterium, and menopause).

## 5. Conclusions

The present study investigated the correlation between parents’ perception and the existence of children’s experience with TKM by analyzing the 2017 national survey of TKM usage. The results indicate that the parents’ experience of using TKM and their awareness contributed to the differences in their children’s experience of using TKM. Our study suggests that the parent’s experience of using TKM could have an impact on the children’s experience of using TKM. In the future, policy-based interventions would have to be considered for the parents when establishing TKM policies for their children.

## Figures and Tables

**Table 1:** Information and characteristics of the respondent’s children.

Category			*n* (%)
Existence of chlidren under 19	Yes		872 (17.4)
	No		4128 (85.6)
TKM experience of children reported by parents	Yes		209 (24.0)
	No		663 (76.0)
Number and TKM experiences of children	Number of children	1	425 (48.7)
	TKM experience of children reported by parents	Yes	78 (18.4)
		No	346 (81.6)
	Number of children	2	393 (45.1)
	TKM experience of children reported by parents	Yes	108 (27.5)
		No	285 (72.5)
	Number of children	3	52 (6.0)
	TKM experience of children reported by parents	Yes	22 (42.3)
		No	30 (57.7)
	Number of children	4	2 (0.2)
	TKM experience of children reported by parents	Yes	1 (50.0)
		No	1 (50.0)

TKM: Traditional Korean Medicine.

**Table 2:** Characteristics of the study population from the survey with and without traditional Korean medicine (TKM) experience of children.

Category		Existence of TKM Experience of Children		Total *n* (%)	χ^2^ (*p*)
		Yes *n* (%)	No *n* (%)		
Parent Gender	Male	57 (27.3)	235 (35.4)	292 (33.5)	4.765 (*p* = 0.029)
	Female	152 (72.7)	428 (64.6)	580 (66.5)	
Residence	Metropolitan	78 (37.3)	293 (44.2)	371 (42.5)	3.793 (*p* = 0.285)
	Chungcheng province	31 (14.8)	85 (12.8)	116 (13.3)	
	Gyeongsang province	55 (26.3)	170 (25.6)	225 (25.8)	
	Jeolla province	45 (21.5)	115 (17.3)	160 (18.3)	
Age	30 s≤	2 (1.0)	31 (4.7)	33 (3.8)	8.196 (*p* = 0.042)
	40 s	79 (37.8)	277 (41.8)	356 (40.8)	
	50 s	111 (53.1)	311 (46.9)	422 (48.4)	
	≥60 s	17 (8.1)	44 (6.6)	61 (7.0)	
Job	Yes	139 (66.5)	449 (67.7)	588 (67.4)	0.107 (*p* = 0.744)
	No	70 (33.5)	214 (32.3)	284 (32.6)	
Household income	Less than 1500 USD	20 (0.5)	7 (1.1)	8 (0.9)	5.036 (*p* = 0.284)
	1500 USD less than 3000 USD	26 (12.4)	105 (15.8)	131 (15.0)	
	3000 USD less than 4500 USD	79 (37.8)	267 (40.3)	346 (39.7)	
	4500 USD less than 6000 USD	73 (34.9)	218 (32.9)	291 (33.4)	
	No less than 6000 USD	30 (14.4)	66 (10.0)	969 (11.0)	
Academic background	Primary or lower school graduate	1 (0.5)	4 (0.6)	5 (0.6)	3.034 (*p* = 0.386)
	Middle school graduate	0 (0.0)	8 (1.2)	8 (0.98)	
	High school graduate	62 (29.7)	210 (31.7)	272 (31.2)	
	University or higher school graduate	146 (69.9)	441 (66.5)	587 (67.3)	
Medical security type	Health insurance (district insurance)	63 (37.3)	149 (22.5)	212 (24.3)	5.619 (*p* = 0.060)
	Health insurance (workplace insurance)	146 (69.9)	512 (77.2)	658 (75.5)	
	Medical care	0 (0.0)	2 (0.3)	2 (0.2)	
Commercial insurance	Subscribed	192 (91.9)	579 (87.3)	771 (88.4)	3.192 (*p* = 0.074)
	Unsubscribed	17 (8.1)	84 (12.7)	101 (11.6)	

**Table 3:** Perception of the survey respondents with and without TKM experience of children.

Variables		Existence of TKM Experience of Children Reported by Parents		Total *n* (%)	χ2(*p*)
		Yes *n* (%)	No *n* (%)		
Parent’s experience on TKM use	Yes	191 (91.4)	477 (71.9)	668 (76.6)	35.220 (*p* < 0.001)
	High school graduate or under	54 (25.8)	154 (23.2)	208 (23.9)	
	University or higher school graduate	137 (65.6)	323 (48.7)	460 (52.8)	
	No	18 (8.6)	186 (28.1)	204 (23.4)	
	High school graduate or under	9 (4.3)	68 (10.3)	77 (8.8)	
	University or higher school graduate	9 (4.3)	118 (17.8)	127 (14.6)	
Parent’s satisfaction on TKM treatment	Satisfied	143 (74.9)	369 (77.4)	512 (76.6)	2.925 (*p* = 0.712)
	High school graduate or under	42 (22.0)	119 (24.9)	161 (24.1)	
	University or higher school graduate	101 (52.9)	250 (52.4)	351 (52.5)	
	Average	47 (24.6)	102 (21.4)	149 (22.3)	
	High school graduate or under	12 (6.3)	34 (7.1)	46 (6.9)	
	University or higher school graduate	35 (18.3)	68 (14.3)	103 (15.4)	
	Unsatisfied	1 (0.5)	6 (1.3)	7 (1.0)	
	High school graduate or under	0 (0.0)	1 (0.2)	1 (0.1)	
	University or higher school graduate	1 (0.5)	5 (1.0)	6 (0.9)	
Parent’s perception on TKM	Well aware	92 (44.0)	234 (35.3)	326 (37.4)	12.125 (*p* = 0.033)
	High school graduate or under	31 (14.8)	71 (10.7)	102 (11.7)	
	University or higher school graduate	61 (29.2)	163 (24.6)	224 (25.7)	
	Average	72 (34.4)	214 (32.3)	286 (32.8)	
	High school graduate or under	19 (9.1)	68 (10.3)	87 (10.0)	
	University or higher school graduate	53 (25.4)	146 (22.0)	199 (22.8)	
	Not sure	45 (21.5)	215 (32.4)	260 (29.8)	
	High school graduate or under	13 (6.2)	83 (12.5)	96 (11.0)	
	University or higher school graduate	32 (15.3)	132 (19.9)	164 (18.8)	
TKM treatment cost	Expensive	135 (64.6)	386 (58.2)	521 (59.7)	6.055 (*p* = 0.301)
	High school graduate or under	46 (22.0)	129 (19.5)	175 (20.1)	
	University or higher school graduate	89 (42.6)	257 (38.8)	346 (39.7)	
	Average	62 (29.7)	239 (36.0)	301 (34.5)	
	High school graduate or under	14 (6.7)	77 (11.6)	91 (10.4)	
	University or higher school graduate	48 (23.0)	162 (24.4)	210 (24.1)	
	Inexpensive	12 (5.7)	38 (5.7)	50 (5.7)	
	High school graduate or under	3 (1.4)	16 (2.4)	19 (2.2)	
	University or higher school graduate	9 (4.3)	22 (3.3)	31 (3.6)	
Intention of re-visit	Yes	198 (94.7)	565 (85.2)	763 (87.5)	14.195 (*p* = 0.003)
	High school graduate or under	61 (29.2)	189 (28.5)	250 (28.7)	
	University or higher school graduate	137 (65.6)	376 (56.7)	513 (58.8)	
	No	11 (5.3)	98 (14.8)	109 (12.5)	
	High school graduate or under	2 (1.0)	33 (3.8)	35 (4.0)	
	University or higher school graduate	9 (4.3)	65 (9.8)	74 (8.5)	
Intention of recommendation	Yes	178 (85.2)	427 (64.4)	605 (69.4)	33.184 (*p* < 0.001)
	High school graduate or under	55 (26.3)	145 (21.9)	200 (22.9)	
	University or higher school graduate	123 (58.9)	282 (42.5)	405 (46.4)	
	No	31 (14.8)	236 (35.6)	267 (30.6)	
	High school graduate or under	8 (3.8)	77 (11.6)	85 (9.7)	
	University or higher school graduate	23 (11.0)	159 (24.0)	182 (20.9)	

**Table 4:** Perception of the TKM effectiveness of diseases of survey respondents with and without TKM experience of children.

Category		Existence of TKM Experience of Children Reported by Parents		Total *n* (%)	χ^2^ (*p*)
		Yes *n* (%)	No *n* (%)		
Disc related disease (herniation of intervertebral disc, spinal stenosis)	Effective	157 (75.1)	480 (72.4)	637 (73.1)	2.364 (*p* = 0.307)
	Ineffective	38 (18.2)	115 (17.3)	153 (17.5)	
	No idea	14 (6.7)	68 (10.3)	82 (9.4)	
Osteoarthritis	Effective	158 (75.6)	474 (71.5)	632 (72.5)	1.754 (*p* = 0.416)
	Ineffective	36 (17.2)	124 (18.7)	160 (18.3)	
	No idea	15 (7.2)	65 (9.8)	80 (9.2)	
Frozen shoulder shoulder pain	Effective	180 (86.1)	550 (83.0)	730 (83.7)	1.221 (*p* = 0.543)
	Ineffective	17 (8.1)	69 (10.4)	86 (9.9)	
	No idea	12 (5.7)	44 (6.6)	56 (6.4)	
Back pain	Effective	191 (91.4)	568 (85.7)	759 (87.0)	5.166 (*p* = 0.076)
	Ineffective	9 (4.3)	58 (8.7)	67 (7.7)	
	No idea	9 (4.3)	37 (5.6)	46 (5.3)	
Sprain	Effective	189 (90.4)	559 (84.3)	748 (85.8)	5.170 (*p* = 0.075)
	Ineffective	11 (5.3)	65 (9.8)	76 (8.7)	
	No idea	9 (4.3)	39 (5.9)	48 (5.5)	
Facial nerve paralysis	Effective	152 (72.7)	480 (72.4)	632 (72.5)	0.112 (*p* = 0.946)
	Ineffective	37 (17.7)	123 (18.6)	160 (18.3)	
	No idea	20 (9.6)	60 (9.0)	80 (9.2)	
Stroke	Effective	132 (63.2)	412 (62.1)	544 (62.4)	0.560 (*p* = 0.756)
	Ineffective	52 (24.9)	180 (27.1)	232 (26.6)	
	No idea	25 (12.0)	71 (10.7)	96 (11.0)	
Hypertension	Effective	58 (27.8)	190 (28.7)	248 (28.4)	0.079 (*p* = 0.961)
	Ineffective	109 (52.2)	339 (51.1)	448 (51.4)	
	No idea	42 (20.1)	134 (20.2)	176 (20.2)	
Diabetes mellitus	Effective	55 (26.3)	157 (23.7)	212 (24.3)	0.699 (*p* = 0.705)
	Ineffective	111 (53.1)	358 (54.0)	469 (53.8)	
	No idea	43 (20.6)	148 (22.3)	191 (21.9)	
Digestive disease	Effective	128 (61.2)	361 (54.4)	489 (56.1)	4.261 (*p* = 0.119)
	Ineffective	59 (28.2)	199 (30.0)	258 (29.6)	
	No idea	22 (10.5)	103 (15.5)	125 (14.3)	
Common cold rhinitis	Effective	124 (59.3)	315 (47.5)	439 (50.3)	9.073 (*p* = 0.011)
	Ineffective	63 (30.1)	249 (37.6)	312 (35.8)	
	No idea	22 (10.5)	99 (14.9)	121 (13.9)	
Dementia	Effective	52 (24.9)	160 (24.1)	212 (24.3)	1.205 (*p* = 0.548)
	Ineffective	113 (54.1)	339 (51.1)	452 (51.8)	
	No idea	44 (21.1)	164 (24.7)	208 (23.9)	
Cancer related pain	Effective	44 (21.1)	149 (22.5)	193 (22.1)	1.461 (*p* = 0.482)
	Ineffective	116 (55.5)	337 (50.8)	453 (51.9)	
	No idea	49 (23.4)	177 (26.7)	226 (25.9)	
Infertility	Effective	72 (34.4)	217 (32.7)	289 (33.1)	0.241 (*p* = 0.887)
	Ineffective	92 (44.0)	296 (44.6)	388 (44.5)	
	No idea	45 (21.5)	150 (22.6)	195 (22.4)	
Skin disease (atopic dermatitis)	Effective	107 (51.2)	250 (37.7)	357 (40.9)	12.043 (*p* = 0.002)
	Ineffective	68 (32.5)	282 (42.5)	350 (40.1)	
	No idea	34 (16.3)	131 (19.8)	165 (18.9)	
Genitourinary disease	Effective	70 (33.5)	188 (28.4)	258 (29.6)	2.104 (*p* = 0.349)
	Ineffective	92 (44.0)	321 (48.4)	413 (47.4)	
	No idea	47 (22.5)	154 (23.2)	201 (23.1)	

## Data Availability

The data will be made available upon reasonable request.
